# Determination of monosaccharide composition in human serum by an improved HPLC method and its application as candidate biomarkers for endometrial cancer

**DOI:** 10.3389/fonc.2022.1014159

**Published:** 2022-11-03

**Authors:** Yulong Chen, Qin Yao, Xuan Zeng, Cui Hao, Xiulian Li, Lijuan Zhang, Pengjiao Zeng

**Affiliations:** ^1^ Department of Gynaecology, The Affiliated Hospital of Qingdao University, Qingdao, Shandong, China; ^2^ Medical Research Center, The Affiliated Hospital of Qingdao University, Qingdao, Shandong, China; ^3^ The Second Affiliated Hospital of Guangzhou University of Chinese Medicine, Guangzhou, China; ^4^ School of Pharmacy, Binzhou Medical University, Yantai, China

**Keywords:** monosaccharide composition, high-performance liquid chromatography (HPLC), serum, biomarker, endometrial cancer

## Abstract

Altered glycan levels in serum have been associated with increased risk of cancer. In this study, we have developed and validated a HPLC-based method to analyze monosaccharide composition (D-mannose, Glucosamine, Galactosamine, Glucuronic acid, D-glucose, D-galactose, D-xylose, L-fucose) in human serum, with L-rhamnose, being used as internal standard. Monosaccharides obtained from hydrolyzed serum samples were derivatized by 1-Phenyl-3-methyl-5-pyrazolone. A ZORBAX XDB-C18 column(150×4.6mm) was used for chromatographic separation with 100 mM ammonium acetate buffer (NH4Ac-HAc, PH=5.5, solvent A), acetonitrile (ACN, solvent B) as a mobile phase. The calibration standard curves for the eight monosaccharides showed good linearity over the range of 2.5-500μg/mL with R^2^ > 0.995. The relative standard deviation values for intra-day and inter-day precision were ≤ 5.49%. Recovery was 69.01-108.96%. We observed that this column exhibited high specificity and selectivity to separate monosaccharides from serum. This method was then applied to quantitatively analyze the serum monosaccharide levels in 30 patients with endometrial cancer and 30 matched healthy controls. Statistical analysis indicated that the serum monosaccharide levels were significantly higher in patients compared with healthy controls (P value< 0.0001). Overall, we report here a simple, reliable, low-cost, and reproducible HPLC method for the separation and quantification monosaccharides in the human serum, which has potential value to serve as a screening marker for endometrial cancer.

## Introduction

The American Cancer Society estimates that 65,950 new endometrial cancer (EC) cases will be diagnosed in 2022 and 12,550 will succumb to this dreadful disease. It is estimated that by 2030, EC will be the third most common cancer affecting American women (1). The main clinical symptom of EC is postmenopausal bleeding (PMB), but only about 5%~10% of PMB cases are diagnosed as EC (2). Premenopausal and perimenopausal women may also present with uncommon symptoms such as increased vaginal discharge and hematuria, which are often ignored by patients thereby significantly reducing the chance of early diagnosis (3). The 5-year survival rate has been reported to be about 74%~91% in early stage (stage I-II) and 57%~66% and 20%~26% in late stage (stage III and IV), respectively (4). It has been reported that early diagnosis can markedly improve the therapeutic effect and prognosis (5). Currently, EC is diagnosed by a combination of transvaginal ultrasound scans (TVUS) and endometrial biopsies, which can cause invasiveness and discomfort in postmenopausal women with a visual analogue scale (VAS) pain score of 6.5 (6, 7). In addition, many technical problems (12~23%) and insufficient tissue quantity (16~68%) in obtaining an endometrial biopsy have also been reported (8). Therefore, it is necessary to develop novel non-invasive blood-based biomarkers, which can significantly improve early diagnosis and outcomes, including survival of EC patients (9).

There is currently no routinely used biomarker in EC patients for diagnostic or prognostic purposes (10). The application of CA125 for recurrent EC is largely limited to late detection, as only 10~20% of women with stage I EC and 25% of women with asymptomatic recurrence display elevated serum CA125 levels (11). In addition, there are number of studies supporting the potential clinical application of Human epididymis protein 4 (HE4) in the diagnosis, prognosis, and monitoring of EC. However, current challenges associated with its application include finding the most appropriate serum cutoff as well as changes in serum HE4 based on the various physiological factors, including age and renal function (12). Although the diagnosis and treatment of EC has now shifted from histological typing to molecular typing, the TransPORTEC and ProMisE systems can stratify the risk based on abnormalities in the POLE and p53 genes in patients, both of which show potential implementation as standard practice for the risk stratification in EC patients. However, both these methods remain underdeveloped, and their potential clinical relevance needs to be further confirmed and validated (13). The above genetic classification is rather complex, and with the development of glycomics technology, glycoprotein biomarkers carrying certain specific glycans have shown better clinical potential, as even small changes in glycosylation might contribute to the tumor development and progression given the widespread nature of glycosylation modifications (14).

Glycosylation is an important form of post-translational modifications (PTMs) found in the proteins (15). The variations in protein glycosylation have been associated with regulation of the formal physiological processes such as aging or further linked with various human diseases, including autoimmune diseases (16, 17), infectious diseases (bacteria, viruses, and parasites) (18–20), and malignancies (21). Aberrant glycosylation is a common phenomenon reported in cellular deterioration and tumor development, and this change often precedes glycoprotein expression (22). For instance, the levels of serum O- or N-glycans are different among the healthy individuals and patients suffering from the different type of cancers (23–26). In addition, different N-glycan structures in serum IgGs that have no direct relationships with cancer cells can be useful distinguish healthy individuals from patients with lung, gastric, prostate, ovarian, and breast cancers (27–31). Moreover, several glycan structural changes have been identified on specific proteins in different types of cancer, such as gastric, nasopharyngeal or endometrial cancer (32), and immunoglobulins or glycoregulatory proteins are aberrantly expressed in EC (33, 34). These published reports clearly indicate the diverse glycan structures, abundantly present in the human blood circulation and tissues, and can potentially serve as promising biomolecules for biomarker discovery (35). However, glycan structure-based biomarkers are difficult to translate into clinical use due to the structural heterogeneity found among patients with different blood types and other technical as well as time-consuming issues (36–38).

**Figure 1:**
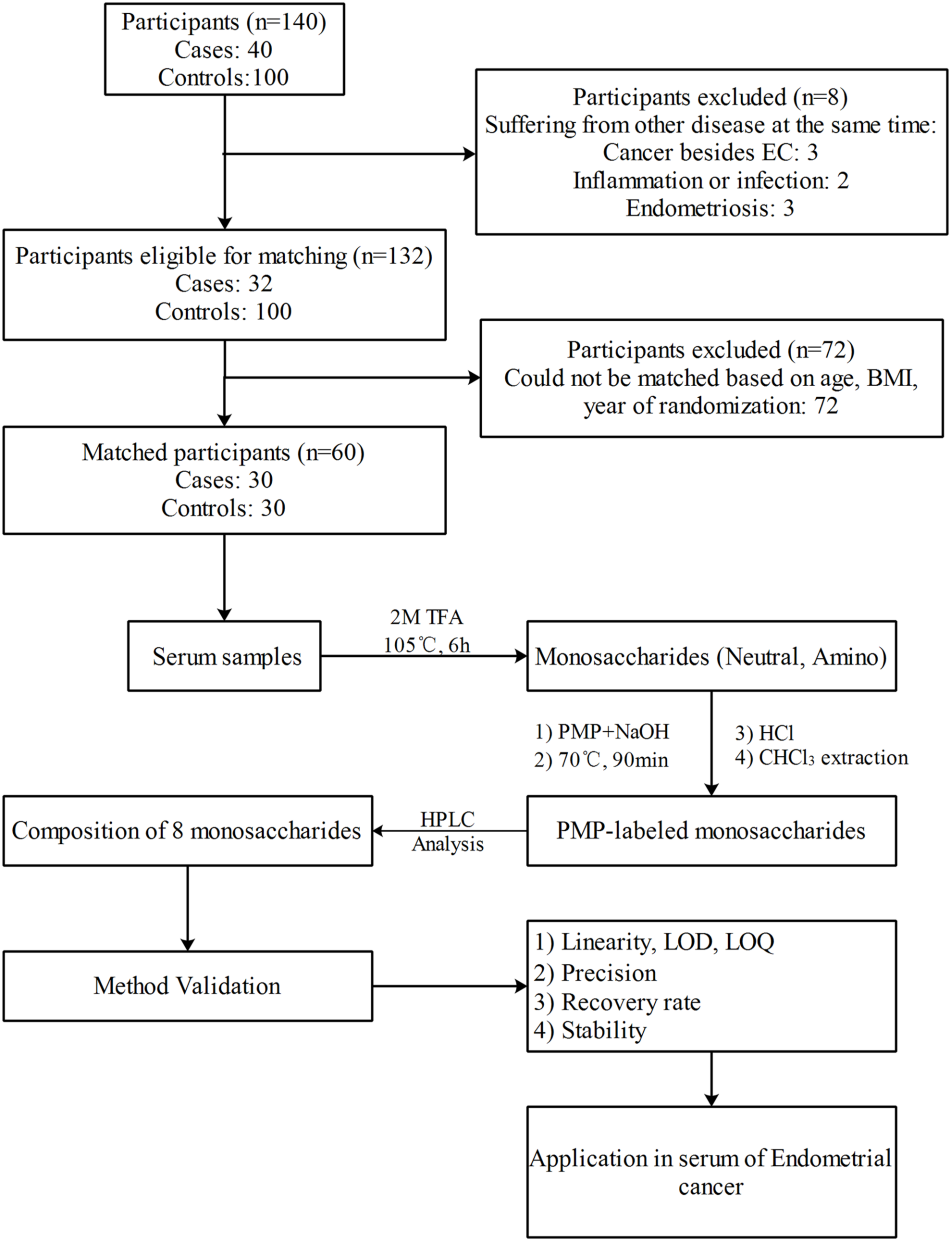
The scheme of the experiment design.

Even though human serum glycan structures have been found to be difficult to characterize (39), they consist of 9 distinct monosaccharides, i.e., D-Glucose, D-galactose, N-Acetyl-D-glucosamine, N-Acetyl-D-galactosamine, L-Fucose, D-Mannose, D-Xylose, D-Glucuronic acid, and N-Acetylneuraminic acid or sialic acid (40). However, few methods have been developed to quantify monosaccharide compositions in the human sera or plasmas for biomarker development. For example, Honda et al. (41, 42) described that commercially available oligosaccharides or glycoprotein could be hydrolyzed to obtain the various component monosaccharides by using an optimized method, which were subsequently labeled with 1-Phenyl-3-methyl-5-pyrazolone (PMP). Based on this, we have developed a reversed-phase high-performance liquid chromatography (HPLC) method for the analysis monosaccharides in the blood of cancer patients. We subsequently found that plasma monosaccharide composition differed in 11 different types of cancer and have successfully applied for a Chinese invention patent (43). In this study, we aimed to develop a simple, reliable, low-cost, and reproducible HPLC method for the separation as well as quantification of monosaccharides in human serum. This method was applied to an exploratory analysis of the serum samples obtained from EC and matched healthy controls to further evaluate its potential role as a biomarker for screening and diagnosis of EC (see **Figure 1**).

## Materials and methods

### Reagents and materials

Monosaccharide standards (≥99.5% purity, Sigma Aldrich, USA), including D-mannose (Man), Glucosamine (GlcN), L-rhamnose (Rha, internal standard, IS), Galactosamine (GalN), Glucuronic acid (GlcA), D-glucose (Glc), D-galactose (Gal), D-xylose (Xyl), L-fucose (Fuc), 1-Phenyl-3-methyl-5-pyrazolone (PMP, Sigma Aldrich, USA) were used for experiments. Hydrochloric acid, Sodium hydroxide, Ammonium acetate, Acetic acid, trifluoroacetic acid (TFA), Ammonia and CHCl_3_ were obtained from Sinopharm Chemical Reagent (Shanghai, China). HPLC-grade acetonitrile and methanol were purchased from Merck, Germany. MilliQ water was used from AQUELIX system (MILLIPORE, Germany). All other chemicals and reagents were of analytical grade and were commercially procured.

### Standard solutions and quality controls

The standard stock solutions of eight monosaccharides (Man, GlcN, GalN, GlcA, Glc, Gal, Xyl and Fuc,10mg/mL) were prepared respectively by dissolving each monosaccharide in water. Standard stock working solution was prepared by mixing the eight monosaccharide stock solutions and diluting them with water to 1mg/mL. The standard curve working solutions were then obtained by making appropriate dilutions of the stock working solution at the concentrations of 500, 250, 100, 50, 25, 10, 5, 2.5, 1, 0.5μg/mL. The low, medium, and high concentration levels of quality control (QC) samples were prepared by applying the same method as the standards at 2.5,25 and 250μg/mL, respectively. An internal standard (IS) solution was prepared by mixing the different reagents consisting of 1mg/L Rha in 2 mol/L TFA as hydrolysis reagent. All standards and samples were filtered through a 0.22μm PVDF syringe filter (Membrane Solutions, TX, USA) before being injected into HPLC system.

### Serum samples

Fasting venous blood was collected from EC patients in the morning before surgery and placed in a sterile procoagulant tube. The serum specimens were collected by centrifugation at 1000 g for 10 min after incubating for coagulation, and then the supernatants were aliquoted and stored at -80°C for further analysis. The serum samples from the matched healthy people were collected in the same way as EC group.

### Monosaccharides preparation

1-Phenyl-3-methyl-5-pyrazolone (PMP)-derivatized monosaccharides were prepared by the method described previously (42)with minor modifications. Briefly, an aliquot (10μL) of serum, standard solution or QCs were mixed with 1 mL of the internal standard solution, respectively. The mixed solution was then incubated at 105°C for 6 h. The resulting reaction solutions were cooled to room temperature and then evaporated to dryness. The residual TFA in the dried samples was further removed in the centrifugal concentrator by adding 200μL methanol to each sample for three times.

Each dried standard, QCs or serum was derivatized with PMP by adding 60μL PMP solution (0.5M dissolved in methanol) and 40μL of 0.3M sodium hydroxide solution, followed by vortexing, and incubation at 70°C for 90 min. The reaction was neutralized by adding 40μL hydrochloric acid (0.3M), and vortexing for at least 5s. The mixtures were then dried in the centrifugal concentrator at 50° C for 30 min. The dried residues were reconstituted in water (70μL) and CHCl_3_ (500μL) and then shaken vigorously. The mixture was then centrifuged at 12,380 g for 10 min, and then the chloroform layer was discarded. This extraction process was performed three times to ensure the complete removal of PMP. Finally, the supernatant was centrifuged at 12,380 g for 10 min and the supernatant was collected for HPLC analysis.

### Chromatographic conditions

The chromatographic fractionation was accomplished by using an Agilent1260 infinity HPLC system (quaternary pump G1311C, high-performance auto-sampler G1367E, thermostatic column compartment G1316A, UV detector DAD, G1315D, Germany) equipped with ZORBAX XDB-C18 column (150×4.6mm, 5μm particle size, Agilent) (44). The separations were performed at 37°C with gradient elution at a flow rate of 1mL/min for 55 min using 100 mM ammonium acetate buffer (NH4Ac-HAc, PH=5.5, solvent A), acetonitrile (ACN, solvent B). The volume of each specimen was 20μL. The applied gradient was then initiated with 0→40→40.1→55min, corresponding to the concentration gradient of B, 15%→22%→15%→15% and detected at λ=245 nm. The peaks were assigned by comparing to a standard mixture of the monosaccharide standards. The relative quantification was conducted using the open-source Agilent Chem Station software Edition B.04.02 SP1 (Agilent, Germany).

### Method validation

Assay validation was conducted according to the FDA guidelines for bioanalytical method validation guidance for industry (45–47). Assay validation was performed with respect to the linearity, limit of detection (LOD), and limit of quantification (LOQ), recovery, precision, and stability.

### Specificity

Specificity refers to the ability of the analytical method used to correctly determine the analyte in the presence of other components (e.g., endogenous substances, degradation products, etc.). A standard concentration of 250μg/ml, internal standard solution and six samples from EC patients and normal serum were mixed and homogenized. Thereafter, the chromatograms of the three groups of samples were compared after HPLC analysis according to the monosaccharides preparation method.

### Linearity of calibration curve

Linearity indicates a proportional relationship between the detection response value and monosaccharide concentration in the sample within the concentration range. The standard curve working solutions mixed with 1 mL of the internal standard solution were measured. Y represented the ratio of monosaccharide peak area A_i_ to IS peak area A_IS_, X represented the ratio of monosaccharide concentration C_i_ to IS concentration C_IS_, and regression calculation IS was carried out by the least square method of “1/X^2^ weighting”. Linear equation and correlation coefficient were recorded. The linearity was considered satisfactory when the correlation coefficients (R^2^) were above 0.99 over the concentration range, linear concentration range should cover the expected concentration range.

### LOD, LOQ and recovery rate

Limit of Detection (LOD) refers to the minimum amount of monosaccharide that can be detected in the sample, Limit of quantification (LOQ) refers to the minimum amount of monosaccharide to be measured in the sample that can be quantitatively detected. Generally, LOD and LOQ are determined by the corresponding concentration when signal-to-noise (S/N) ratio of 3 and10 respectively has been reached.

To examine the recovery rate, six different batches of QC samples, mixed serum (six patients and healthy controls) and QC samples added into the mixed serum were prepared, respectively. The three group samples were analyzed according to the monosaccharide preparation method, and the mean peak areas of each monosaccharide in the three groups were labeled as A, B, and C, respectively. According to the mean peak area, the recovery of Man, GlcN, GalN, GlcA, Glc, Gal, Xyl and Fuc was calculated according to the formula:


Recovery rate % = C/(A + B) × 100%

### Precision

Quality control samples 2.5, 25 and 250μg/mL were treated according to the monosaccharide preparation method. Each concentration was analyzed in parallel for five different times. The actual concentration of the quality control samples was calculated according to the mean standard curve on that very day. During the three days of methodological investigation, the above procedures were performed every day. The precision RSD of the assay was calculated, the intra-day precision was obtained by comparing the data from one day, and finally the inter-day precision and accuracy were calculated.

### Stability

**Stock solution stability.** To determine the stability of the stock solution, the stock solutions of different dates were also compared: one was freshly prepared whereas the other was stored at 4°C for two months. The experiment was carried out in triplicates.

**Freeze–thaw stability.** QC samples prepared in mixed human serum after 3 cycles of freezing (-80°C) and thawing (room temperature) were compared with the freshly prepared samples at the same concentration. The experiment was carried out in triplicates.

**Short-term (bench-top) stability.** QC samples in the mixed human serum were freshly prepared and left on the bench-top at room temperature for 24 h. All the samples were compared with the freshly prepared samples at the same concentrations. Experiments were conducted in three times.

**Processed samples stability.** The stability of the treated samples was determined by comparing the freshly obtained serum extracts with the remaining serum extracts in the automatic sampler at the room temperature for 24 h. The experiment was carried out in triplicates.

**Long-term storage stability.** QC samples of the monosaccharide in mixed human serum were prepared and stored at -80°C for 2 weeks. All the samples were compared with the freshly prepared samples of the same concentration. The experiment was carried out in triplicates.

### Assay application in quantification of clinical human serum samples

This validated HPLC method was applied to quantify the monosaccharide concentrations in a panel of 60 serum samples from EC patients (n = 30) and matched healthy controls (n = 30). Informed consent was obtained from each patient included in the study. The study protocol conforms to the ethical guidelines of the 1975 Declaration of Helsinki and was conducted in accordance with the guidelines set by the Ethics Committee of the Affiliated Hospital of Qingdao University and the samples were subjected to the informed consent procedure before collection and signed a written informed consent form with the patients.

Inclusion criteria: All the participants were those who visited the Affiliated Hospital of Qingdao University. All EC patients included in the study group were subjected to the clinical and pathological review, from first onset of symptoms to hospitalization in the Department of Obstetrics and Gynecology who had not received chemotherapy or radiotherapy before recruitment. During the same period, healthy individuals who matched age and menopausal status of the study group, displayed no signs or symptoms of endometrial disease and normal pelvic color ultrasound findings served as the control group. Exclusion criteria (1): Diagnosed with other benign and malignant tumors; (2) Preoperative chemoradiotherapy and targeted therapy; (3) anemia patients with blood transfusion therapy and oral iron therapy; (4) Diagnosed with other systemic diseases, with incomplete medical records; (5) Preoperative signs of acute and chronic infection; (6) Diagnosed with other gynecological diseases, pregnancy diseases, lactating pregnant women.

### Statistical analysis

All the data was analyzed with SPSS statistical software (version 23.0; SPSS Inc., Chicago, Illinois, USA), GraphPad Prism 8.0 (GraphPad Software Inc., San Diego, CA, USA). We used the multi-sample Shapiro-Wilk test to examine the normality of continuous variables. The continuous variables have been presented as mean ± standard deviation or median (25th percentile, 75th percentile). Student’s t test (normal distribution) or Mann-Whitney U test (skew distribution) was used to compare the difference of continuous variables and p-values<0.05 were considered as statistically significant.

## Results and discussion

### Chromatography

An equal concentration mixture of eight different monosaccharides (Man, GlcN, GalN, GlcA, Glc, Gal, Xyl, and Fuc) were labeled with PMP and separated by reverse-phase HPLC. All the PMP-labeled monosaccharides were separated with a baseline retention time of at least 1 min. Inter-run reproducibility regarding retention times was observed to be very good for all the monosaccharides. The peaks of all monosaccharides were sharp and symmetric, resolutions >1.5. Since Rha is not reported to be present in the glycoproteins and other glycoconjugates, it was included in the mixture as an internal standard to facilitate the sample quantification. During acid hydrolysis, N-acetylglucosamine and N-acetyl galactosamine were found to lose their acetyl groups, generating glucosamine and galactosamine, Glucuronic acid and xylose were destroyed to varying degrees (42). Sialic acid is an acid-labile ketose that was destroyed during hydrolysis and its content was not detected.

**Figure 2:**
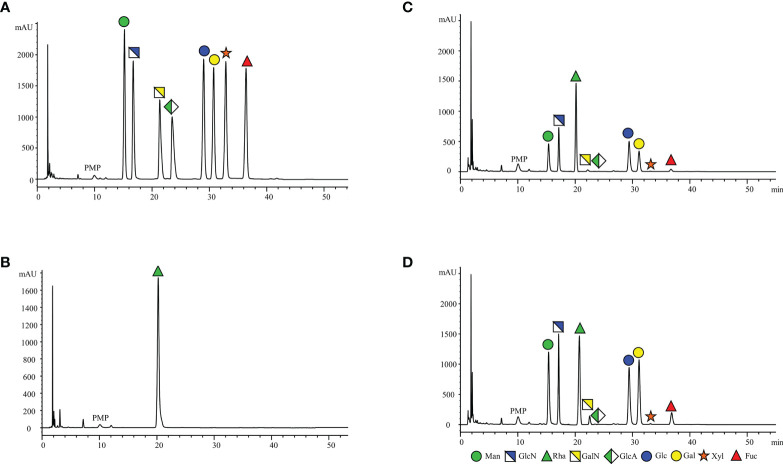
Representative HPLC chromatograms of PMP-labeled monosaccharides. **(A)** monosaccharides standards (250μg/mL); **(B)** Internal standard; **(C)** Healthy control and **(D)** EC patient serum sample with the internal standard. (1) Man (RT: 15.37 ± 0.05 min), (2) GlcN (RT: 17.15 ± 0.04 min), (3) Rha (RT: 20.32 ± 0.09 min), (4) GalN (RT: 22.08 ± 0.06 min), (5) GlcA (RT: 24.27 ± 0.05 min), (6) Glc (RT: 29.53 ± 0.06 min), (7) Gal (RT: 31.25 ± 0.07 min), (8) Xyl (RT: 33.33 ± 0.07 and min), (9) Fuc (RT: 36.83 ± 0.07 min).

In addition, the comparison of **Figures 2A–D** revealed that no distinct interfering peaks appeared at the corresponding retention times of the eight monosaccharides and the internal standard, thus indicating that the endogenous substances of the serum species did not directly interfere with the determination of the eight monosaccharides and the internal standard rhamnose. **Figure 2** shows the chromatogram representatives from: (A) Monosaccharide standards (250μg/mL); (B) Internal standard; (C) Healthy control and (D) EC patient serum sample with the internal standard.

### Linearity, LOD and LOQ

The linearity of the calibration curves was established over the concentration range of 2.5-500μg/mL with the correlation coefficient values R^2^ >0.995. The LOD and LOQ for each of the eight monosaccharides were in the range of 0.74−1.24μg/mL and 1.98−3.74μg/mL, respectively. The linear range, correlation coefficient, LODs, and LOQs of all analytes have been summarized in **Table 1**.

**Table 1:** Analytical performance of the method.

Monosaccharide	Retention Time (min)	Correlation coefficient R²	Concentration range (μg/mL)	LOD(μg/mL)	LOQ(μg/mL)
Man	15.37 ± 0.05	0.9999	2.5-500	0.85	1.98
GlcN	17.15 ± 0.04	0.9995	2.5-500	0.88	2.19
GalN	22.08 ± 0.06	0.9998	5-500	0.91	2.79
GlcA	24.27 ± 0.05	0.9994	5-500	1.24	3.74
Glc	29.53 ± 0.06	0.9998	2.5-500	0.86	2.26
Gal	31.25 ± 0.07	0.9991	2.5-500	0.87	2.27
Xyl	33.33 ± 0.07	0.9982	2.5-500	0.78	2.10
Fuc	36.83 ± 0.07	0.9993	2.5-500	0.74	2.14

### Precision and recovery rate

The intra- and inter-day precision and relative recovery of eight monosaccharides at three concentration levels have been shown in **Table 2**. The intra-day precision (coefficient of variation, CV) of the developed method was calculated and found to be over the range of 0.01−5.49%, whereas the inter-day precision (CV) ranged between 0.0007and 4.18%. These results demonstrated the acceptable reproducibility of the absolute peak area, thus indicating that the precision of the method was satisfactory. The method also depicted acceptable accuracy with the recovery values of three concentration levels of QC samples ranged from 69.01−108.96% for all the eight monosaccharides. Precision and recovery were all found to be within the acceptable range, thereby indicating that the current method was reliable and reproducible.

**Table 2:** Recovery rate, and precision of the method in quality controls at low, medium, and high concentrations.

Monosaccharide	Precision (CV/%) intra-day (n=5)			Precision (CV/%) inter-day (n=5)			Recovery rate %		
	Low	Medium	High	Low	Medium	High	Low	Medium	High
Man	1.99	2.24	0.69	1.67	3.10	0.57	101.36	105.63	98.12
GlcN	0.16	0.92	0.67	2.25	3.69	0.43	103.44	105.11	99.78
GalN	1.68	0.24	0.12	2.44	3.45	0.93	105.44	108.96	101.36
GlcA	1.38	3.84	5.49	1.23	2.59	1.17	77.73	77.06	69.01
Glc	1.02	1.49	0.42	2.87	2.93	0.88	101.52	102.52	93.01
Gal	3.99	1.88	0.01	2.23	3.12	0.22	101.96	105.51	97.90
Xyl	4.31	1.70	1.91	4.18	3.92	0.0007	81.44	87.86	79.54
Fuc	0.53	1.78	1.66	3.66	3.66	0.01	98.21	104.36	89.53

### Stability

**Stock solution stability.** It was found that compared to the samples obtained from freshly prepared stock solution, those obtained from the stock solution stored at 4°C for 2 months displayed 81.88-100.01% (**Table 3**), thus indicating that the monosaccharides stock solution was stable at 4°C for a period of at least 2 months.

**Table 3:** Results of Stability study of monosaccharides in human spiked serum.

Monosaccharide	Stock solution stability	Freeze-thaw stability/%			Short-term (bench-top) stability/%			Processed sample stability/%			Long-term storage stability/%		
		Low	Medium	High	Low	Medium	High	Low	Medium	High	Low	Medium	High
Man	89.84	97.29	98.85	101.21	100.05	106.17	100.42	99.97	99.67	101.84	106.65	98.14	101.70
GlcN	86.95	91.85	100.97	100.86	99.75	98.08	99.15	100.38	101.32	101.61	100.99	94.93	95.40
GalN	91.21	102.65	101.15	99.94	99.86	97.80	101.27	100.21	101.97	101.62	103.77	100.63	97.46
GlcA	100.01	100.86	92.26	103.30	92.83	101.22	99.25	115.78	143.95	120.42	86.29	80.30	77.11
Glc	91.26	94.21	103.16	101.32	98.60	103.69	97.91	100.85	101.33	103.08	108.30	97.80	103.79
Gal	89.21	97.50	99.98	101.13	101.36	102.82	96.87	100.23	100.77	101.87	95.90	95.24	96.50
Xyl	84.24	103.43	115.00	98.89	105.21	109.08	94.49	100.20	101.75	102.64	100.77	102.57	107.73
Fuc	81.88	101.43	113.55	99.40	99.38	108.26	96.61	98.61	98.90	102.59	97.18	98.69	103.45

**Freeze-thaw stability.** This group of QC samples prepared in the combined human serum showed an average of eight monosaccharides level of 97.89-105.77%, thus indicating that eight monosaccharides in the human serum were stable even after three freeze-thaw cycles.

**Short-term (bench-top) stability.** The stability of eight monosaccharides of the three groups of QC samples in the mixed human serum was 97.77-102.92%, respectively. This data indicated that eight monosaccharides in the human serum could be stabilized at room temperature for at least 24 h on the laboratory bench.

**Processed samples stability.** The serum extracts left on the workbench at room temperature for 24 h showed an average of 100.03-126.71% eight monosaccharides compared to the freshly prepared serum extracts, thus indicating that treated monosaccharides serum extracts were stable at the room temperature for at least 24 h.

**Long term storage stability.** QC samples from this group of mixed human serum showed an average remaining monosaccharides level of 81.24-103.69%, thus indicating that monosaccharides in the human serum was stable for at least 14 days at -80°C.

All validation results demonstrated that the established method was sufficiently linear, sensitive, precise, recovery and stability for the intended monosaccharide analysis.

### Application in Serum of Endometrial cancer patients and healthy controls

In this study, we have analyzed the serum monosaccharide composition of 30 primary EC patients and 30 matched healthy controls using the developed method (**Table 4**). We observed that Man, GlcN, Glc, and Gal were the four most abundant structures and effectively constituted most of the monosaccharide pool. The four monosaccharides GalN, GlcA, Xyl, and Fuc were detected in small amounts in the serum samples. GlcA was destroyed to varying degrees below the limit of quantitation during the hydrolysis procedure. The serum expression levels of these six monosaccharides (Man, GlcN, GalN, Glc, Gal, and Fuc) were found to be significantly higher in cases in comparison to controls. Fuc was expressed at 3.6-fold higher levels in EC patients than in controls, about 3.0-fold in Man and Gal, and about 2.0-fold in GlcN and GalN and Glc, whereas only Xyl expression levels were significantly lower in EC patients than in controls (P value<0.0001), respectively.

**Table 4:** Monosaccharide concentrations in endometrial cancer patients.

Monosaccharide	Healthy (n=30, Mean (SD), μg/mL)	EC (n=30, Mean (SD), μg/mL)	P-value
Man	42.85 (8.45)	127.7 (33.52)	<0.0001
GlcN	112.6 (14.30)	220.6 (47.35)	<0.0001
GalN	7.81 (1.48)	13.48 (1.85)	<0.0001
GlcA^#^	0.26 (0.04)	1.03 (0.05)	<0.0001
Glc	96.99 (20.38)	206.1 (61.28)	<0.0001
Gal	38.5 (6.53)	111.1 (27.40)	<0.0001
Xyl	3.34 (0.29)	2.67 (0.42)	<0.0001
Fuc	3.89 (0.89)	14.21 (4.07)	<0.0001

## Discussion

The process of finding confounder insensitive and diagnostically reliable biomarkers of EC cancer is of huge importance to clinical care. Glycans are the most abundant and structurally dynamic biomolecules, which have been found to be structurally different in physiological and pathological conditions (48). Thus, serum glycans have been recognized as attractive biomolecules for biomarker discovery. However, over 30 clinically used serum cancer biomarkers have relatively low sensitivity and specificity due to their limited glycan information contents (39, 49). Currently, nearly all prior studies related to the serum glycans as possible cancer biomarkers have been primarily focused on resolving complicated serum glycan structures by using complicated preparation procedures and expensive instrumentations, such as LC-MS, which makes it difficult to translate promising research results into clinical applications. Thus, we adopted a novel approach to simplify the serum glycan information into quantitative monosaccharide compositions.

The bottleneck of our approach was to optimize the serum glycan hydrolysis conditions. In our previous study, we have used a Picotag station to optimize acid hydrolysis conditions for glycosaminoglycans (50–52). By using the Picotag station-based hydrolysis condition, we found that the serum GlcN and GalN concentrations in patients with lung cancers were significantly higher as compared to the healthy individuals and the same assay was also used to detect the presence of contaminant in heparin (50). Since no Picotag station was available in China, we have optimized a workable hydrolysis condition for analyzing the serum or plasma samples. Moreover, even after using the optimized acidolysis condition, we found that Sialic acid was destroyed, followed by varying degrees of loss of GlcA and Xyl. Thus, further optimizing acid hydrolysis condition is desirable for further clinical applications.

Our study has several advantages for blood circulation-based biomarker development. First, our method included a simple PMP-labeling procedure, a clean chromatograph with baseline separation of all the monosaccharides, good linearity, precision, recovery, and stability. Second, this method can be readily implemented in any laboratory with a typical HPLC equipment. Third, only 10μL of serum from either EC patients or healthy individuals were used for the assay without any other manipulations. Fourth, the data of eight monosaccharides from the serum sample were obtained simultaneously from a single HPLC profile, which can effectively generate quantitative data report for each patient. Moreover, we have found based on the results of our prior studies that monosaccharide composition of hydrolyzed serum glycans could serve as biomarkers for colorectal cancer (53). Thus, to further understand the molecular mechanisms, we have employed tumor and non-tumor mouse models to identify the potential source of abnormal serum glycans in different diseases (54). We found that livers and spleens but not tumors in the mouse model were primarily responsible for the increased monosaccharide concentrations of hydrolyzed plasma glycans (55).

In conclusion, we have developed a simple, reliable, low-cost, and reproducible HPLC method for the separation and quantification monosaccharides in the human serum, which may be suitable for clinical specimen analysis. To our knowledge, this is the first individual HPLC method developed and applied to quantify the concentration levels of eight monosaccharide compositions in the clinical human serum samples. The findings of this study provided preliminary evidence that EC patients have significantly elevated levels of monosaccharides in addition to xylose in comparison to the healthy controls, thereby suggesting that the serum levels of monosaccharide composition might prove to be useful markers for conducting the screening and diagnosing the disease. These results are promising, but further validation studies in larger and independent series of patient samples are warranted and are currently ongoing.

## Data availability statement

The original contributions presented in the study are included in the article/Supplementary Material. Further inquiries can be directed to the corresponding authors.

## Ethics statement

Informed consent was obtained from each patient included in the study. The study protocol conformed to the ethical guidelines of the 1975 Declaration of Helsinki and was conducted following the guidelines set by the Ethics Committee of the Affiliated Hospital of Qingdao University. The patients/participants provided their written informed consent to participate in this study.

## Author contributions

PZ and LZ conceived and guided the research project. PZ and YC carried out the experiments. All authors participated in the sample collections, data analysis, and data interpretation. PZ, LZ, and YC wrote the manuscript. All authors contributed to the article and approved the submitted version.

## Funding

This research was supported by the Natural Science Foundation of Shandong Province, China (Grant No. ZR2021QH207, ZR2021QH167) the National Natural Science Foundation of China (Grant No. 81672585), the Taishan Scholar Fellowship to LZ.

## Acknowledgments

This work was supported by the Department of Gynecology in the Affiliated Hospital of Qingdao University, for the blood sample collection and the gynecologists provide with clinical diagnosis.

## Conflict of interest

The Affiliated Hospital of Qingdao University has filed patent applications on the basis of this work.

The remaining authors declare that the research was conducted in the absence of any commercial or financial relationships that could be construed as a potential conflict of interest.

## Publisher’s note

All claims expressed in this article are solely those of the authors and do not necessarily represent those of their affiliated organizations, or those of the publisher, the editors and the reviewers. Any product that may be evaluated in this article, or claim that may be made by its manufacturer, is not guaranteed or endorsed by the publisher.

## Abbreviations

ACN, Acetonitrile; DAD, Diode array detector; EC, Endometrial cancer; Fuc, Fucose; Gal, Glucosamine; GalN, Galactosamine; Glc, Glucose; GlcA, Glucuronic acid; GlcN, Glucosamine; HE4, Human epididymis protein 4; HPLC, High Performance Liquid Chromatography; IS, Internal standard; LC-MS, Liquid Chromatography couple Mass Spectrometry; LOD, Limit of detection; LOQ, Limit of quantitation; Man, Mannose; PMP, 1-Phenyl-3-methyl-5-pyrazolone; Rha, Rhamnose; RSD, Relative standard deviation; RT, Retention time; TFA, Trifluoroacetic acid; Xyl, Xylose.
